# Rainwater isotopes in central Vietnam controlled by two oceanic moisture sources and rainout effects

**DOI:** 10.1038/s41598-020-73508-z

**Published:** 2020-10-05

**Authors:** Annabel Wolf, William H. G. Roberts, Vasile Ersek, Kathleen R. Johnson, Michael L. Griffiths

**Affiliations:** 1 Department of Geography and Environmental Sciences, Northumbria University, Newcastle-upon-Tyne, NE1 8ST UK; 2 Department of Earth System Science, University of California, Irvine, CA 92697 USA; 3 Department of Environmental Science, William Paterson University, Wayne, NJ 07470 USA

**Keywords:** Atmospheric science, Hydrology, Palaeoclimate

## Abstract

The interpretation of palaeoclimate archives based on oxygen isotopes depends critically on a detailed understanding of processes controlling the isotopic composition of precipitation. In the summer monsoonal realm, like Southeast Asia, seasonally and interannually depleted oxygen isotope ratios in precipitation have been linked to the summer monsoon strength. However, in some regions, such as central Vietnam, the majority of precipitation falls outside the summer monsoon period. We investigate processes controlling stable isotopes in precipitation from central Vietnam by combining moisture uptake calculations with monthly stable isotope data observed over five years. We find that the isotopic seasonal cycle in this region is driven by a shift in moisture source from the Indian Ocean to the South China Sea. This shift is reflected in oxygen isotope ratios with low values (− 8 to − 10‰) during summer and high values during spring/winter (0 to − 3‰), while 70% of the annual rainfall occurs during autumn. Interannual changes in precipitation isotopes in central Vietnam are governed by the timing of the seasonal onset and withdrawal of the Intertropical Convergence Zone, which controls the amount of vapour contributed from each source.

## Introduction

Tracing the hydrological cycle with stable isotopes in precipitation has greatly advanced our understanding of climatic processes, due to their different behaviour during evaporation and condensation^1–3^. Stable isotopes in precipitation are also extensively used as palaeoclimate proxies, for example when recorded in the stable isotopic composition of tree ring cellulose^4,5^, leaf waxes^6^, ice cores^7,8^, and speleothems^9–16^. However, processes controlling the isotopic composition of rainfall vary strongly in space^17–20^ and time^21–23^. This represents a particular challenge for palaeoclimate reconstructions. The key for interpreting the palaeoclimatic signal in water isotopes is understanding which of these processes are the most important, and this can be quantified by looking at stable isotopes in modern rainfall. Traditionally, variability in the isotopic composition of tropical rainfall was explained by an inverse relationship between the local precipitation amount and the isotope ratio ($\delta ^{18}O_p$), termed the ’amount effect’^2^. It was later found that this relationship is much more complex and a variety of controlling processes explains the strong spatial and temporal variability in between sites. Seasonal changes in tropical rainwater isotopes have been attributed to changes in moisture source location and moisture history^18,24^, atmospheric processes, such as rainout along the travel path^14,22^, and changes in convection strength^19^. It has been shown that the precipitation type (e.g. convective versus stratiform^25^), cloud-top height^26^ and local microphysical processes within the cloud, such as condensation rates, re-evaporation and crystallization processes^27,28^, can also have a role on the variability of the isotope ratio in tropical rainfall^20^.

**Figure 1:**
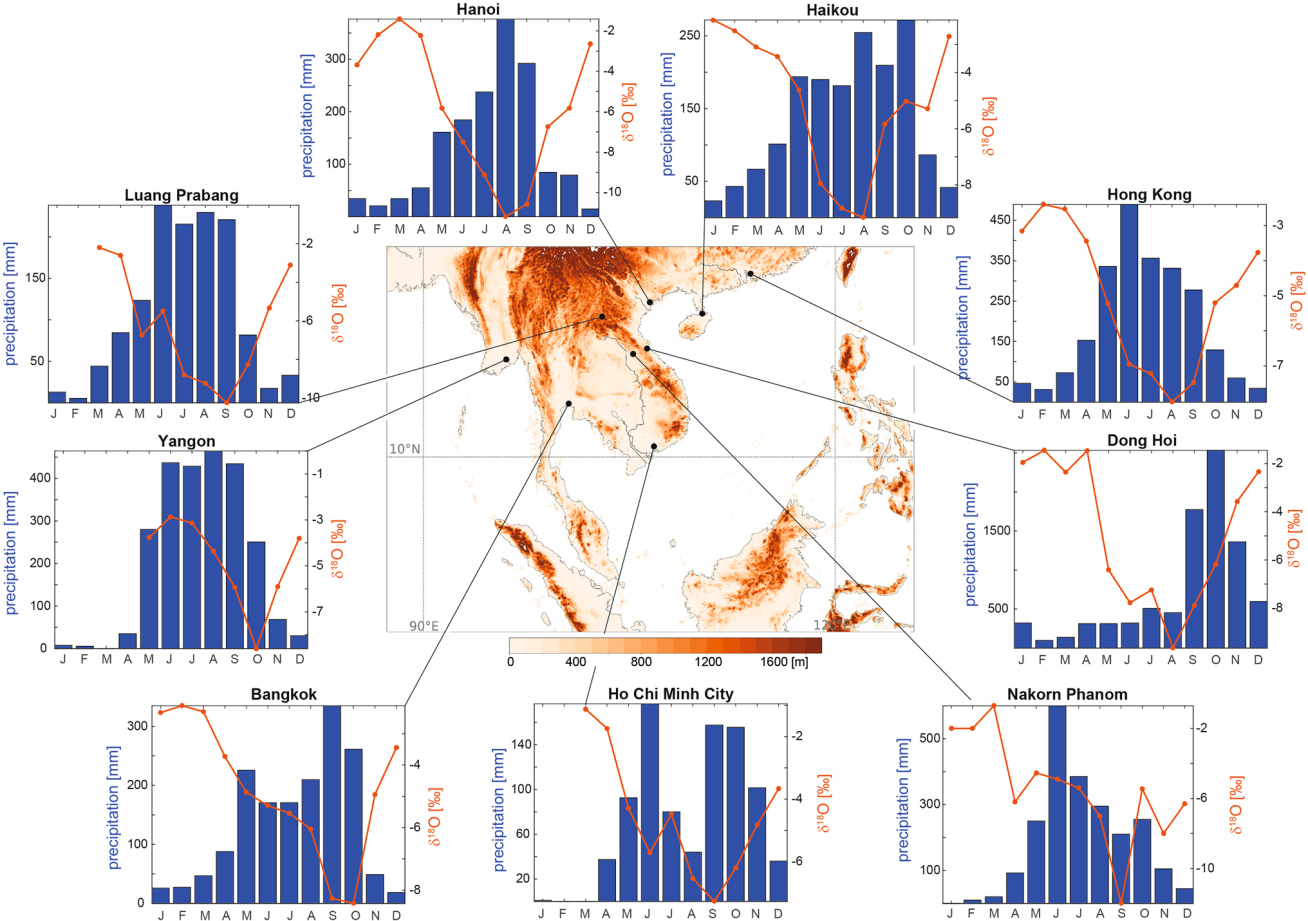
Map showing local topography^29^, location and multi-year average of precipitation and $\delta ^{18}O_p$ for Global Network of Isotopes in Precipitation (GNIP) stations^44^ in East and Southeast Asia. Data for Ho Chi Minh City from Le Duy et al.^30^ and Munksgaard et al.^31^ and for Nakorn Phanom from Noipow^32^. Central Vietnam’s boreal autumn rainfall regime is unique in the region and is represented by the Dong Hoi station. The map was generated using the Matplotlib Basemap Toolkit version 1.1.0 (https://matplotlib.org/basemap/index.html).

Understanding these processes is particularly challenging in regions with sparse and short records of rainfall monitoring, such as Southeast Asia. Thus far, seasonal variability in rainwater isotopes from Southeast Asia has been linked to source location^24,33,34^, regional and local rainfall amount^34,35^, convection strength^19^, and rainout along the moisture advection path^30,35^. Research has mainly focused on the isotopic composition of the summer monsoon compared to the winter season, since the shift in $\delta ^{18}O_p$ between summer and winter is largest. However, it remains unclear what drives the seasonal cycle in $\delta ^{18}O_p$ throughout the entire year, especially for sites with a rainy season independent of the summer monsoon. The focus on these two seasons is problematic for mainland Southeast Asia, where the timing of peak rainfall varies strongly across the peninsula and is not restricted to strong summer monsoon rainfalls^36^ (Fig. 1). For example, annual rainfall in central Vietnam is strongly affected by tropical cyclones that peak during autumn (September to November) and rainfall associated with northeasterly winds^37,38^ (Fig. 1). This makes central Vietnam ideal to investigate the impact of non-summer monsoonal rainfall on the seasonal cycle in rainwater isotopes.

Previous modelling studies have identified the topography of mainland Southeast Asia as a crucial factor in modulating the timing of its rainfall season^39,40^. Autumn rainfall in central Vietnam is a direct consequence of local topography interacting with the annual migration of the Intertropical Convergence Zone (ITCZ), creating zones of intense rainfall and rain shadows depending on the season^40^. An inverse relationship between topography and the isotopic composition of rainfall has long been observed and is described as the ’altitude effect’^41^. However, the altitude effect is less pronounced in the tropics^42^ and its relative importance is disputed^43^. Mainland Southeast Asia has two main mountain ranges located between Myanmar and Thailand to the west (the Tenasserim Hills), and between Laos and Vietnam to the east (the Truong Son Mountains or Annamite Mountains). Therefore, central Vietnam is ideal to investigate the importance of these effects on stable isotopes in precipitation there.

In this study, we combine modelling of monthly moisture uptake locations for precipitation arriving in central Vietnam and monthly stable isotope ratios collected over five years. We aim to identify controls on the seasonal cycle in precipitation isotopes from central Vietnam to understand processes at work and identify the primary controls on seasonal variability independently from the summer monsoon season. Further, we investigate the impact of rainout effects using a spatial array of precipitation isotope data from five sites in mainland Southeast Asia. Then we explore interannual variability of precipitation isotopes in order to estimate the potential of stable isotopes from central Vietnam for palaeoclimate reconstructions.

## Results and discussion

**Figure 2:**
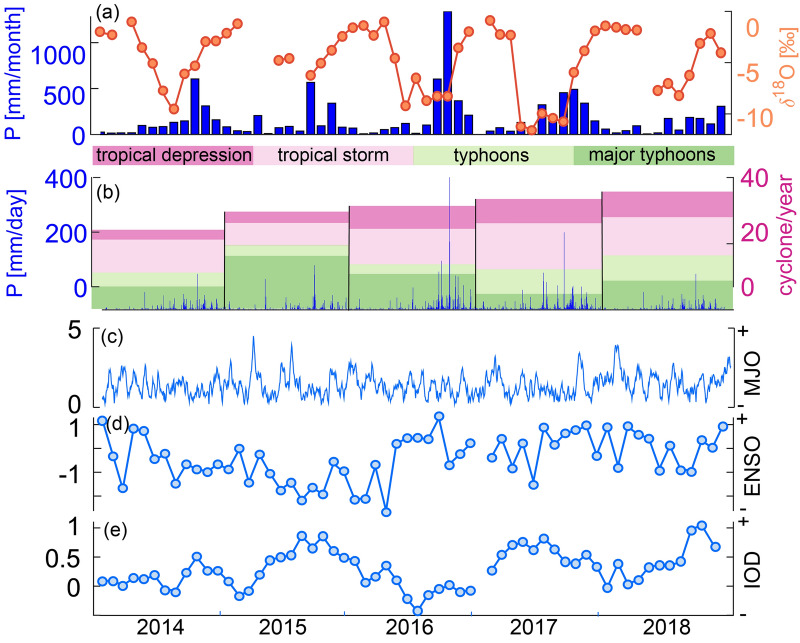
Comparison of (**a**) $\delta ^{18}O_p$ and total monthly precipitation at Dong Hoi and (**b**) daily precipitation at Dong Hoi and the number of tropical cyclones^45^ in the western North Pacific, where the colours indicate the number of occurrences within each class. (**c**) shows the Madden-Julian Oscillation (MJO), based on the Real-time Multivariate MJO Index^46^ (**d**) El Niño–Southern Oscillation (ENSO), retrieved from the Southern Oscillation Index^47^ (**e**) the Indian Ocean Dipole (IOD), retrieve from the Dipole Mode Index^48^, for the years 2014 to 2018.

Between 2014 and 2018 monthly $\delta ^{18}O_p$ values from the Dong Hoi GNIP station^44^ show a distinct seasonal cycle, with lowest values from May until September ($-\,7.8 \pm 1.2$‰) and highest $\delta ^{18}O_p$ values ($-\,1.9 \pm 0.4$‰) during December to April (Fig. 2). This seasonal pattern is similar to the isotopic composition of rainfall in southern China (Hong Kong)^26^ and Hanoi (Fig. 1). Following removal of the seasonal cycle, we found no significant correlation ($\hbox {r}^2 = 0.12$, p-value $= 0.02$) between monthly oxygen isotopes and precipitation amount in central Vietnam, therefore factors other than the amount effect must be invoked to explain the seasonal $\delta ^{18}O_p$ variability. Further, there is no correlation between the monthly oxygen isotopes and the number of tropical cyclones occurring in the western North Pacific ($\hbox {r}^2 = 0.0002$, p-value $= 0.91$). To investigate the influence of changes in the moisture source location on rainfall in central Vietnam, we simulate the monthly moisture pathways in the following section.

### Moisture source location and large-scale circulation

**Figure 3:**
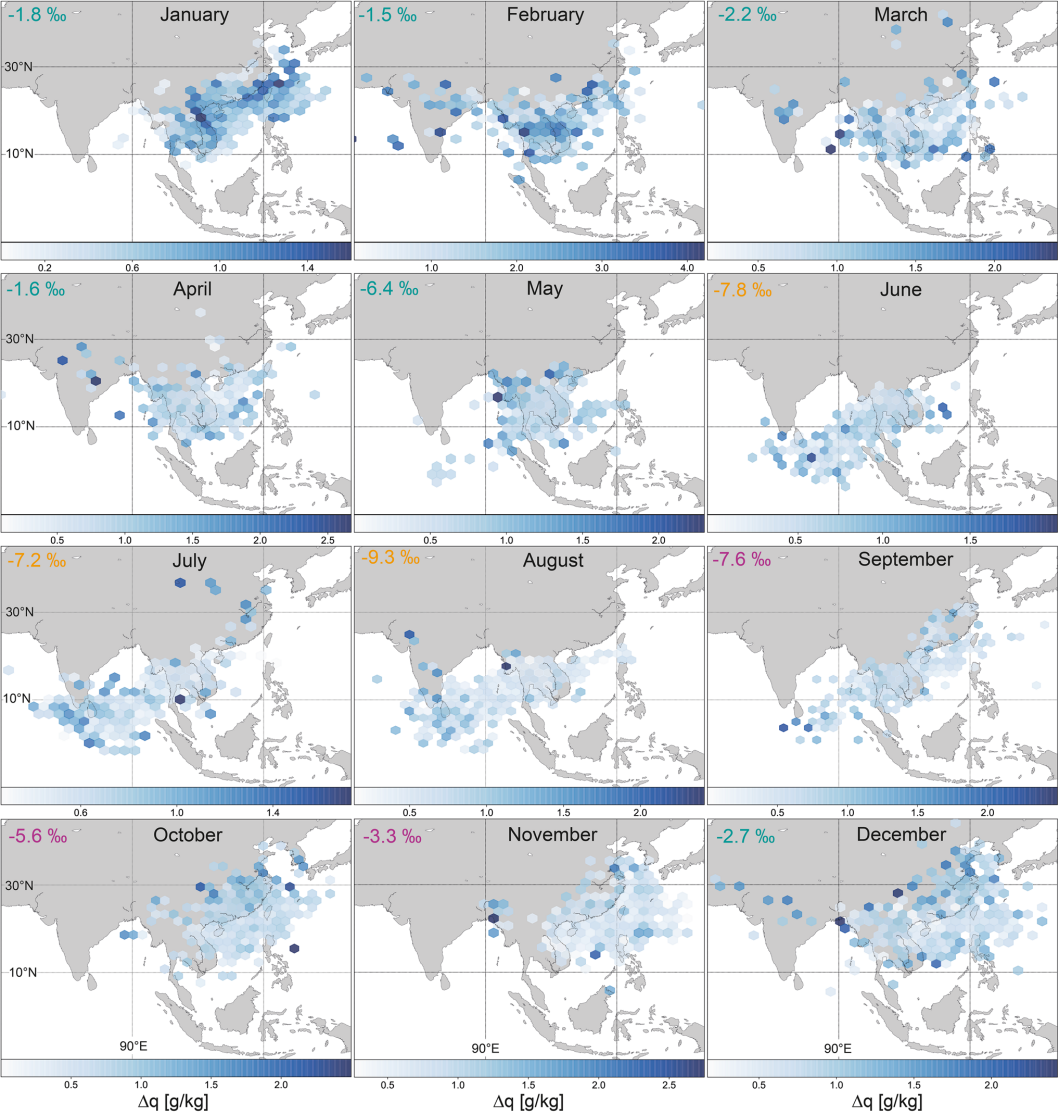
Monthly moisture uptake locations for the years 2014 to 2018 shown as hexagonal binning, with the mean value of $\Delta \hbox {q}$ occurring in each cell. $\Delta \hbox {q}$ describes the changes in specific humidity of an air parcel between two set time intervals. Areas with no colour show that no trajectory was projected by HYSPLIT for this point. The calculation is based on 6-hourly backward trajectories for each month, and reveals a seasonal shift in moisture source location between the period of June to August and of September to May. The monthly $\delta ^{18}O_p$ values are indicated in the upper left corner of each map. The maps were generated using the Matplotlib Basemap Toolkit version 1.3.0 (https://matplotlib.org/basemap/index.html).

 There are three main air mass sources for mainland Southeast Asia: the Indian Ocean, the Pacific Ocean, and continental Asia^30,33,34^. Calculating the air mass trajectories using the Hybrid Single-Particle Lagrangian Integrated Trajectories (HYSPLIT) model helps to understand the general air movement; however, it is of limited use for identifying the location of moisture uptake^49^. Primary evaporation can occur anywhere along the trajectory path, leaving the trajectory analysis with a given uncertainty. We therefore calculate the monthly moisture uptake based on the specific humidity to estimate the uptake location for moisture arriving in central Vietnam using PySplit^50^. The analysis shows that from June to August, most moisture is taken up from the southern Bay of Bengal and during the rest of the year from the northern South China Sea (Fig. 3). During May and September rainfall originates from both sources, suggesting the switch from one source to the other occurs during these months.

**Figure 4:**
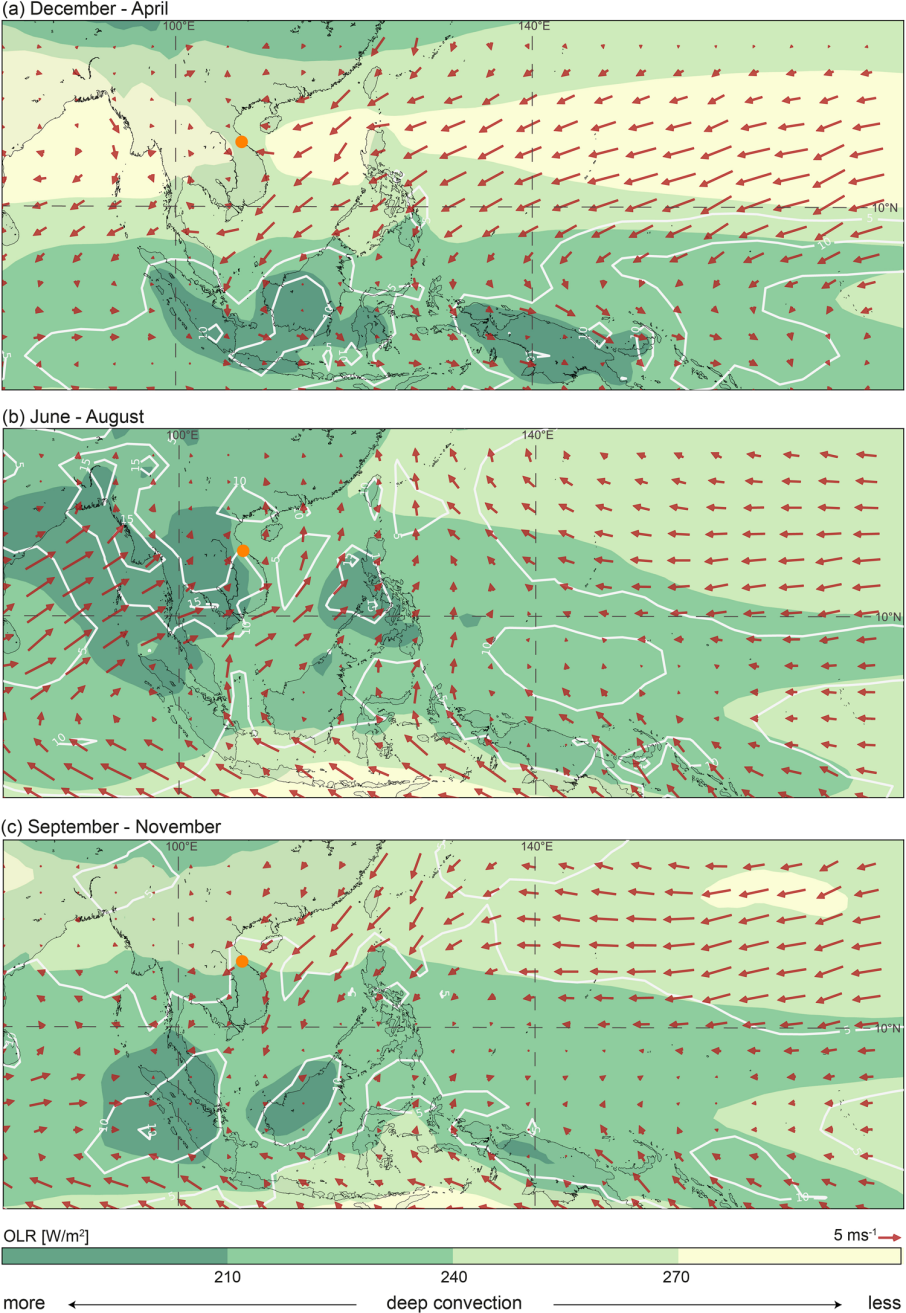
Surface climatology (**a**) December to April, (**b**) June to August and (**c**) September to November. Surface wind (monthly average of 10 m u and v component downloaded from the ERA5 dataset^56^) (red arrows), monthly mean Interpolated Outgoing Longwave Radiation (OLR) data^57^ (coloured contours) and Global Precipitation Climatology Centre (v2018)^58^ monthly precipitation data (white contours plotted at 5, 10, 15 mm day$^{-1}$). The location of Dong Hoi is represented by the orange dot. The maps were generated using the Matplotlib Basemap Toolkit version 1.1.0 (https://matplotlib.org/basemap/index.html).

The change in moisture source location is linked to the seasonal migration of the ITCZ and the associated large-scale atmospheric circulation changes. The ITCZ in the West Pacific is located around $5 \,^{\circ }\hbox {S}$ during December to February and north of $15\, ^{\circ }\hbox {N}$ in June to August^51^ (Fig. 4). During April, the ITCZ begins to move northwards from near the Equator crossing southern Vietnam in early May^52^. In mid-May, the ITCZ moves rapidly northwards within a few days^53^, explaining the southwesterly winds, which increase the contribution of moisture to mainland Southeast Asia from the Bay of Bengal. This effect is clearly visible in central Vietnam as a shift in the moisture uptake location towards the Indian Ocean during May (Fig. 3). The Indian Ocean remains the main moisture source location throughout summer (June to August) and mid-September (Fig. 4b). Beginning in September, the ITCZ slowly retreats southwards resulting in northeasterlies bringing intense rainfall into central Vietnam at this time^54,55^. Our moisture uptake analysis for central Vietnam shows this large-scale change in circulation pattern with a high contribution of moisture from the northern South China Sea from mid-September onwards (Fig. 3). As the ITCZ continues to move south during October to December, easterlies weaken and moisture is taken up more locally from mainland Southeast Asia and part of the South China Sea.

**Figure 5:**
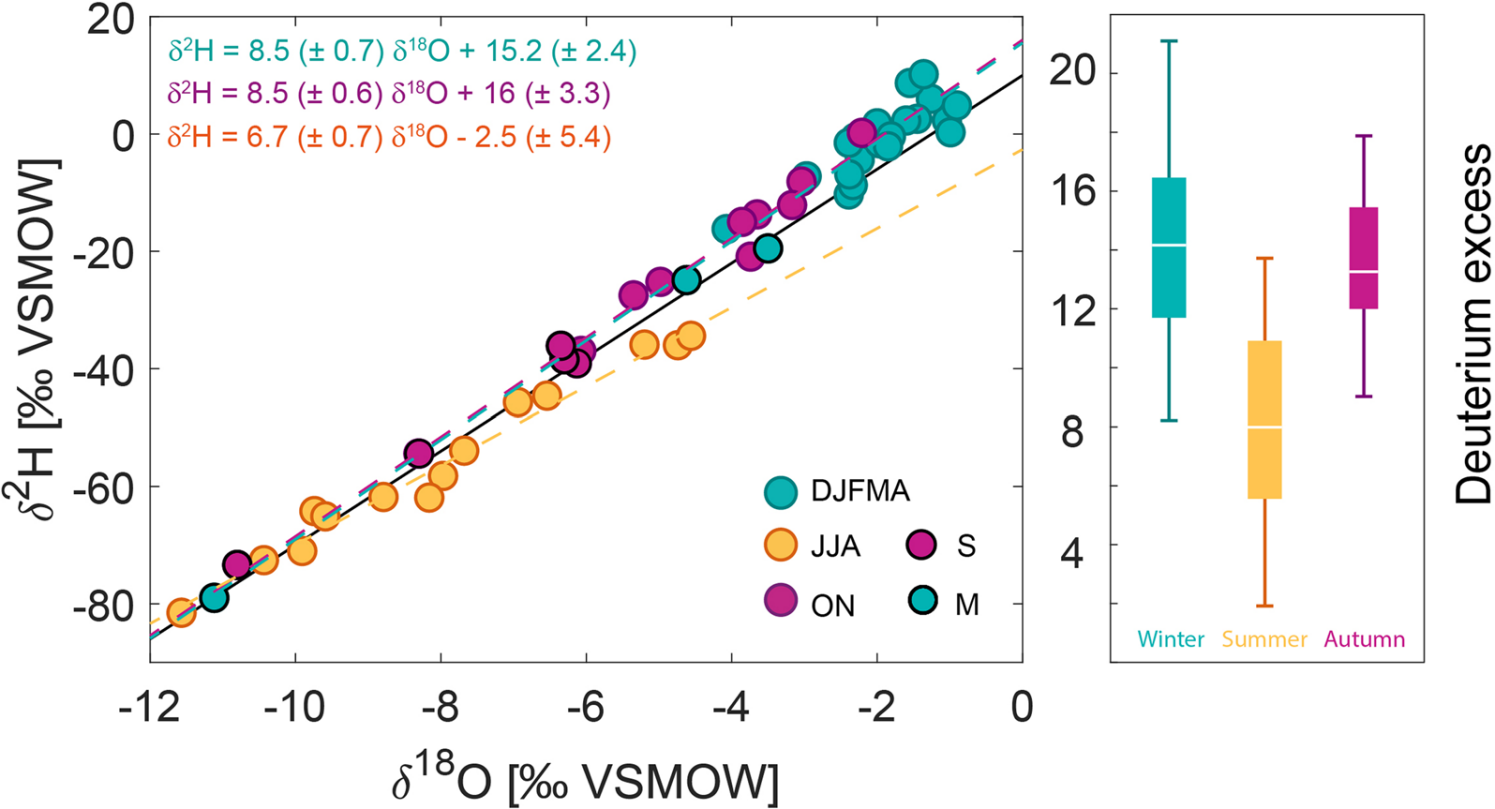
Local Meteoric Water Line (LMWL) in central Vietnam for September, October and November (purple), December to May (turquoise) and June to August (yellow). Since September and May are the transition months where the moisture source changes, they are highlighted with a black outer circle. Monthly data points for the deuterium excess are coloured according to each season. The Global Meteoric Water Line (GMWL) is colour-coded in black and local meteoric water lines are colour-coded according to each season. Deuterium excess values are lower during summer and higher during winter/spring. This is emphasised in the box-and-whisker plot on the right, showing that summer strongly deviates from the rest of the year.

Our findings are supported by the deuterium excess (Fig. 5) defined as: d-excess (‰) = $\delta ^2\hbox {H - }8 \times \delta ^{18}\hbox {O}$^59^. The deuterium excess is a result of the different fractionation rates of oxygen and hydrogen isotopes during kinetic fractionation^60^. During kinetic fractionation, hydrogen fractionation is faster than oxygen fractionation and the rate of fractionation depends on relative humidity at the evaporation site^61^. Kinetic fractionation occurs mainly during evaporation, rather than condensation^61^. Therefore, the deuterium excess acts as a fingerprint of climatic conditions during primary evaporation, independent of rainout processes along the travel path^60^. Drier source regions with low relative humidity are associated with an increased deuterium excess and vice versa^62^. In our data, the deuterium excess in summer rainfall has lower values compared to winter and spring rainfall. Deuterium excess values of autumn rainfall clusters in between (Fig. 5). This confirms our previous results based on moisture source location and shows that the deuterium excess reflects two oceanic sources: the Indian Ocean during summer with most of the values around 5–10‰ and Pacific Ocean during spring, autumn and winter when most values range around 11–17‰. Further, the increased deuterium excess during winter reflects the lower relative humidity in the South China Sea during this time. The relative humidity in the Bay of Bengal is high during summer due to the strong convection related to the Indian Summer Monsoon, which is reflected by low deuterium excess values (Fig. 5).

**Figure 6:**
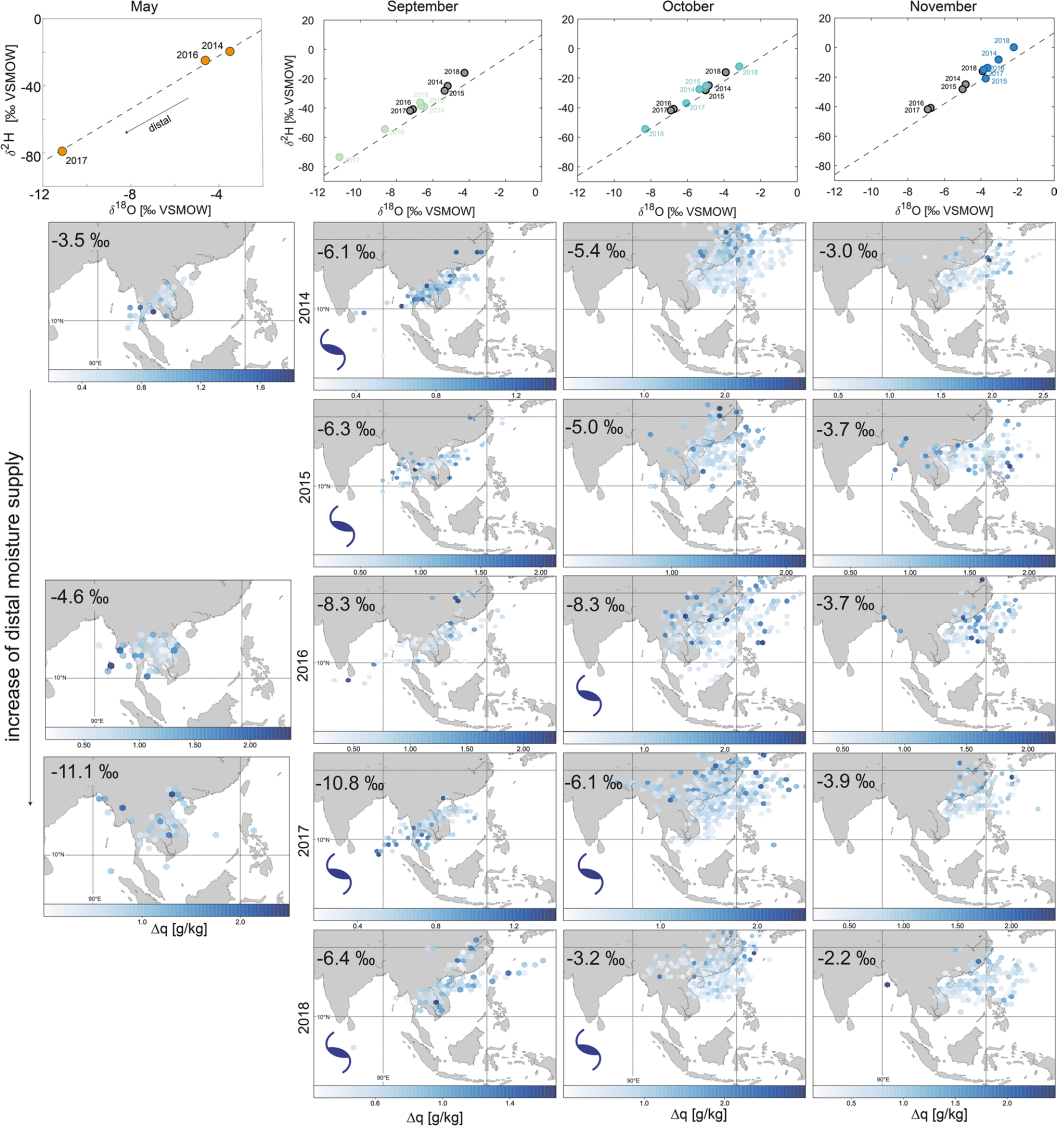
Plots $\delta ^2\hbox {H} - \delta ^{18}\hbox {O}$ for May (orange), September (green), October (turquoise) and November (blue), with the annual means of September, October and November in grey. Grey dashed line shows the GMWL. The maps show hexagonal binning of moisture uptake for each month from 2014 to 2018. Months with tropical cyclones^45^ that made landfall^63^ in central Vietnam are highlighted with a dark blue whirl symbol. The maps were generated using the Matplotlib Basemap Toolkit version 1.3.0 (https://matplotlib.org/basemap/index.html).

We now consider in more detail the isotopic variability of rainfall during the transition periods between moisture sources in May and September (Fig. 3), when the deuterium excess shows a large variability (Fig. 5). The deuterium excess depends on the relative contribution from each source, which can vary from year to year. Rainfall from May 2017 is strongly depleted, which is caused by generally larger range of moisture source regions compared to 2014 (Fig. 6 left column). Lower values in 2017 can be linked to an increased contribution of more distal moisture sources compared to 2014 and 2016. This is a first hint that interannual variability of oxygen isotopes in rainfall can be linked to source location.

Additionally, the link between source location and interannual variability is evident during September to November rainfall (Fig. 6). Considering the averaged values for this time, two clear clusters for 2014/15 and 2016/17 are evident (Fig. 6). This relation is reflected in the source location (Fig. 6 middle columns). In 2016 and 2017, moisture arrives from a more distal location, such as near Sri Lanka and the Gulf of Thailand (Fig. 6). This is not the case in 2014/15 and 2018, when moisture arrives from the proximal eastern Bay of Bengal and off the coast from northern and central Vietnam (Fig. 6). This interannual variability in moisture source is related to the timing in the withdrawal of the ITCZ from Vietnam^64^. An early withdrawal (2014/15) causes the formation of an anti-cyclonic circulation in the South China Sea, partly blocking the south-westerly flow from bringing moisture from the Bay of Bengal to central Vietnam. This leads to more positive deuterium excess. A late withdrawal enables more moisture contribution from the southern Bay of Bengal (south of Sri Lanka) and the Gulf of Thailand (Fig. 6)^40,65^, leading to more negative deuterium excess values. Several studies highlighted that tropical cyclones affect interannual autumn rainfall variability in central Vietnam^30,37,66^, thus it is of importance to consider tropical cyclone activity as a potential factor in modulating $\delta ^{18}\hbox {O}$. Tropical cyclones can result in unusually low $\delta ^{18}\hbox {O}$ values in related rainfall^67^. Using the Joined Typhoon Warning Center annual reports^45^, we identify tropical cyclones, that made landfall in central Vietnam during autumn 2014 to 2018 (Fig. 6). In general, the numbers of tropical cyclones in the western North Pacific show an increasing trend over the years from 2014 to 2018 (Fig. 2). In our dataset, we identify cyclones in September 2014, September 2015, October 2016, September and October 2017 and September and October 2018 (Fig. 6).The only September during our data period without a cyclone is 2016. Rainfall in September 2017 has the lowest $\delta ^{18}\hbox {O}$ value compared to other years, which can be explained by the strong contribution of moisture from the Bay of Bengal (Fig. 6). A similar pattern is evident for September 2016. Therefore, low $\delta ^{18}\hbox {O}$ is not necessarily the result of tropical cyclone activity. During October 2014 to 2018, there is no moisture source in the Bay of Bengal, rather moisture was sourced from the South China Sea. The Octobers with lowest $\delta ^{18}\hbox {O}$ values (October 2016 and 2017) appear to have received moisture from a more distal source, north of the Philippines. However, the difference in distal source contribution appears to be limited. In October 2016 the typhoon Aere brought strong rainfall into central Vietnam, which resulted in the highest recorded rainfall amount in our dataset (Fig. 2). We cannot exclude the possibility that typhoon Aere contributed to the depleted $\delta ^{18}\hbox {O}$ in October 2016, however, this is a single event and does not allow for further investigation from a monthly dataset. October 2018 shows that moisture is contributed mainly from a proximal source, which potentially explains the high $\delta ^{18}\hbox {O}$ values during this year, despite the potential influence of typhoon Yutu. In the months of May and November no tropical cyclones made landfall in central Vietnam during the time-span of our dataset.

Several studies linked the seasonal cycle in rainwater isotopes in Asia to a shift from oceanic to continental sources including Southeast Asia^24,33^, Taiwan^68^, Thailand^34^ and southern China^69^. Our findings suggest that in central Vietnam $$\delta ^{18}O_p$$ is controlled by a shift between two oceanic sources, rather than a shift between a continental and oceanic source (Fig. 3). Establishing the Local Meteoric Water Line (LMWL) is a useful tool to investigate the contribution of continental moisture or secondary evaporation along the travel path. Plant transpiration returns the water vapour with nearly no change in deuterium excess, whereas open water or soil evaporation leads to an increased deuterium excess and sub-cloud evaporation to a decreased deuterium excess^70^. This means that an increased deuterium excess represents an increased continental moisture contribution and a decreased deuterium excess indicates secondary evaporation. The LMWL for central Vietnam is: $$\delta ^2\hbox {H}$$$$=$$ (8.6 ± 0.3‰) $$\times$$$$\delta ^{18}\hbox {O}$$ + (15 ± 2‰) with an $$\hbox {r}^2$$ of 0.98 (n = 54) calculated following Sachs et al.^71^. We calculate the LMWL for each season and find that during autumn, spring and winter, the LMWL slope is increased by 0.5‰ and the intercept by 5.6‰ compared to the GMWL (Fig. 5). This deviation suggests a limited contribution of continental moisture (lake, river or soil moisture) from mainland Southeast Asia during this time of the year^70^. During summer the LMWL has a slope of 6.7, which is less than 8 of the GMWL and an intercept of $$-\,2.5 \pm 5.4$$‰ (Fig. 5). This decrease again suggests a limited influence of continental evaporation. The depletion in the deuterium excess could be a result of re-evaporation of raindrops after condensation (sub-cloud evaporation)^2,60,70^.

### Spatial variability of $\delta ^{18}O_p$ across mainland Southeast Asia

 Thus far, we have focused on explaining the mechanisms controlling the temporal variability of rainwater isotopes in central Vietnam. However, we have not explained the spatial evolution, nor fully considered processes occurring during condensation, rather than evaporation. Investigating how rainwater isotopes vary along the moisture travel path, is useful to understand the importance of rainout processes. These effects are strongest when moisture travels via landmasses and for central Vietnam this is the case during summer (June to August). To explore the spatial variability of $\delta ^{18}O_p$ in mainland Southeast Asia during this time, we investigate the effects of local topography and rainout. Rayleigh fractionation due to rainout occurs not only while moisture travels landwards, but also due to orographic uplift^41^. During summer, air masses reaching central Vietnam have a western source and cross two mountain ranges before reaching our study site^72^, thus making this time ideal to understand these processes.

**Figure 7:**
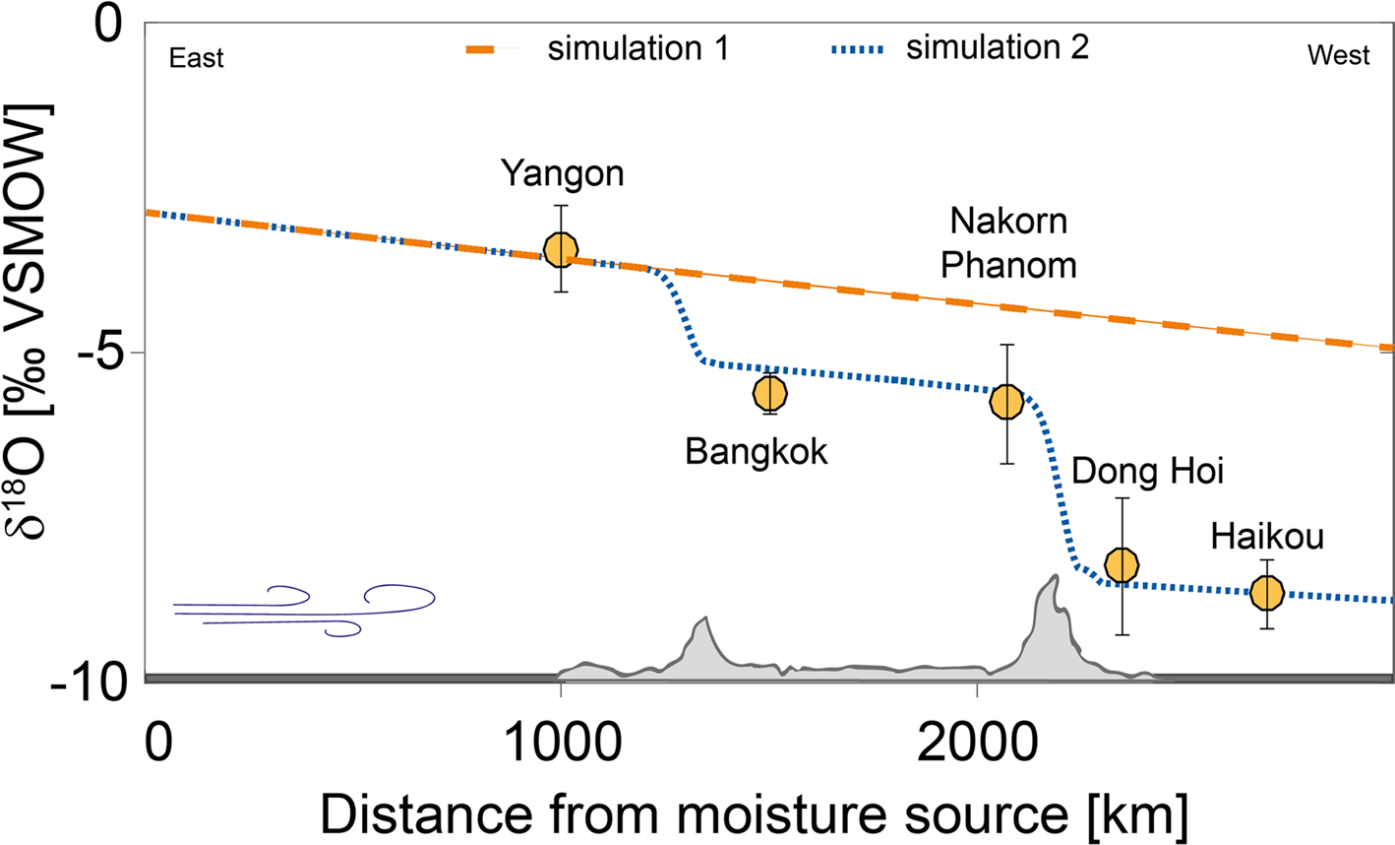
The spatial evolution of oxygen isotopes in precipitation over mainland Southeast Asia. Simulation 1 (orange line) simulates mainland Southeast Asia as a flat surface. Simulation 2 (dashed blue line) includes local topography. Observed long-term means of GNIP data are shown in yellow, with error bars indicating the standard deviation in oxygen isotopes for each location.

We perform two simulations: one with mainland Southeast Asia being represented as a flat land surface (Simulation 1) and one considering local topography to understand the effects of rainout and orographic uplift (Simulation 2). Simulation 1 (Fig. 7) models the rainout in water isotopes from the Bay of Bengal to Hainan (Haikou) and predicts a rainout effect of − 0.68‰ per 1000 m. Considering the GNIP $\delta ^{18}O_p$ data across the transect, it is clear that the rainout effect alone cannot correctly explain this spatial variability. Simulation 2 shows that the spatial development of $\delta ^{18}O_p$ along the transect is better simulated when topography is included in the model (Fig. 7). We simulate the effects of the Tenasserim Hills (700 m elevation in the simulation), positioned between Myanmar and Thailand and of the Truong Son Mountains (1500 m elevation in the simulation), located between Laos and Vietnam. The simulation predicts a negative shift in oxygen isotopes of − 1.44‰ for the Tenasserim Hills and of − 2.89‰ for the Truong Son Mountains, which is an isotopic lapse rate of − 0.3‰/100 m and − 0.2‰/100 m, respectively. These values are within typical rates for the altitude effect of − 0.15 to − 0.5‰ per 100 m^73^. This simulation is in good agreement with observed data (Fig. 7) and shows that the altitude effect alone can explain 70% of the spatial variability in rainwater isotopes during summer. During the summer months, the vertical temperature gradient is greater compared to winter, amplifying the altitude effect during this time^68^. The altitude effect has also been shown to be larger during the rainy season, related to stronger rainout^42^. While the dry season is during summer in central Vietnam, Fig. 4 shows that this is not the case for the rest of the Peninsula, which undergoes the rainy season during summer. Sturm et al.^42^ simulated the altitude effects in tropical South America and found that, during the rainy season, more than 60% of the $\delta ^{18}O_p$ variance is related to altitude according to observed data, which is similar to our results. Our simulations suggest that the rainout and altitude effects combined can explain the spatial variability of oxygen isotopes in mainland Southeast Asia during summer. However, the relation between $\delta ^{18}O_p$ and altitude in mainland Southeast Asia needs more detailed investigation, ideally using isotope-enabled general circulation models. Our results are preliminary and hint towards the importance of orography and local advection for the spatial evolution of water stable isotopes.

### Implications for palaeoclimate research

**Figure 8:**
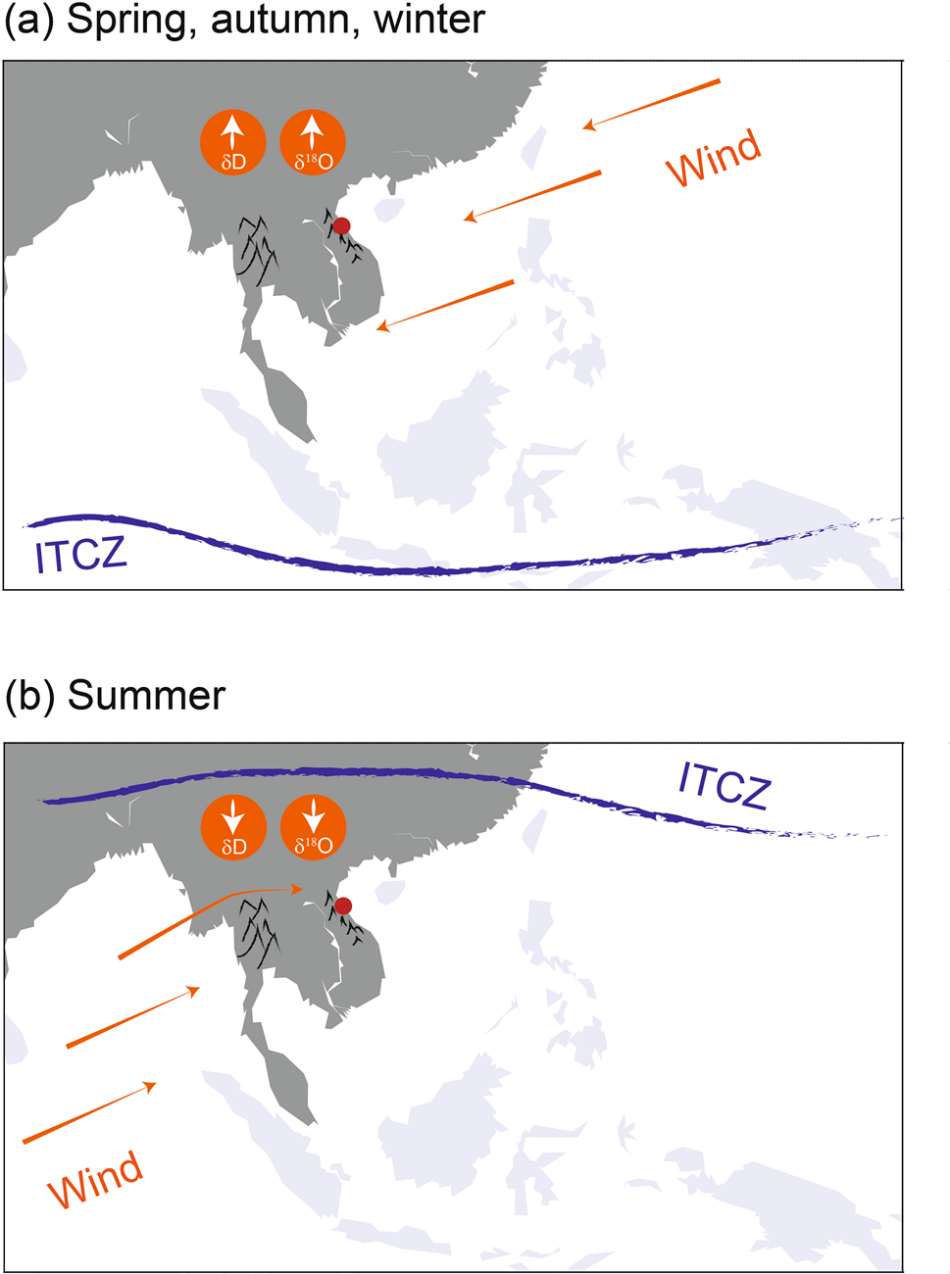
Schematic explaining general circulation pattern (orange), oxygen isotopes ($\delta ^{18}\hbox {O}$) and deuterium excess ($\delta D$) in central Vietnam reacting to the ITCZ position (blue) during (**a**) spring, autumn and winter and (**b**) summer. The maps were generated using the Matplotlib Basemap Toolkit version 1.1.0 (https://matplotlib.org/basemap/index.html).

 The seasonal variability of rainwater isotopes in central Vietnam is controlled by a seasonal shift between two moisture source locations: the Bay of Bengal and the South China Sea. This shift in source location is regulated by the position of the ITCZ (Fig. 8). The timing of the seasonal migration determines the duration of each source’s moisture contribution, thus the ITCZ is controlling the year to year variability in average $\delta ^{18}O_p$. Hence, we show that $\delta ^{18}O_p$ in central Vietnam is sensitive to small-scale variations in the position of the ITCZ. This offers the opportunity for palaeoclimate archives, such as speleothems and tree ring cellulose, to use changes in $\delta ^{18}O$ as indicators of ITCZ dynamics, especially for periods where large changes have been inferred^74–77^.

Further, variability in the ITCZ position is tightly linked to broader tropical ocean dynamics including ENSO and the IOD^78^. This link is mainly established via variability in the local Hadley circulation, which is modulated by changing sea surface temperatures (SSTs) in the western Pacific and thus ENSO^79^. An extensive body of literature based on tree ring cellulose $\delta ^{18}\hbox {O}$^4,5,80–83^ and $\delta ^{18}\hbox {O}$ in precipitation^34,35^ has confirmed the importance of SST variability in the Pacific and Indian Oceans for $\delta ^{18}O_p$ in mainland Southeast Asia. Tree ring cellulose $\delta ^{18}\hbox {O}$ from Thailand^81^, Laos^5^, Cambodia^83^ and Vietnam^4^ shows a clear link between $\delta ^{18}\hbox {O}$ and ENSO, with El Niño events leading to higher $\delta ^{18}\hbox {O}$ and La Niña to lower $\delta ^{18}\hbox {O}$ values. This relation can be enhanced or reduced by modulations of Indian Ocean SSTs^35,82^. In addition, positive $\delta ^{18}\hbox {O}$ anomalies during June to September in rainfall from Laos have been linked to more proximal moisture sources from the Bay of Bengal^35^. This pattern is comparable to an early ITCZ withdrawal in our $\delta ^{18}\hbox {O}$ dataset, showing more proximal sources in the Bay of Bengal as well. These similarities suggest a link between interannual variability in $\delta ^{18}O_p$, and the ITCZ position and ENSO events across mainland Southeast Asia. This means that palaeoclimate records from central Vietnam based on $\delta ^{18}O_p$ can potentially be used to reconstruct regional climate dynamics related to ocean SST anomalies and the evolution of ITCZ through time.

In addition to $\delta ^{18}O_p$ variability across mainland Southeast Asia, studies also investigated the effects of ENSO and the IOD on local rainfall amount. They found a strong spatial variability in the relation of SST anomalies and rainfall anomalies across the Peninsula^36^ and throughout the seasons^84^. This emphasises that rainfall amount and $\delta ^{18}O_p$ do not necessarily correspond, since the impact of ENSO on $\delta ^{18}O_p$ appears to be consistent across the Peninsula, whereas ENSO induced rainfall variability is spatially heterogeneous^85^. Therefore, $\delta ^{18}O_p$ from mainland Southeast Asia can potentially be used to trace large-scale circulation patterns, like shifts in the ITCZ and ENSO, while proxies for local rainfall can be used to investigate how these climate drivers control the local hydroclimate. However, our dataset is limited in time and restricts any long-term observations.

## Conclusion

The timing of the rainy season in central Vietnam differs strongly from the rest of mainland Southeast Asia, with peak rainfall from September to November. Therefore, central Vietnam offers the opportunity to investigate the seasonal evolution of stable isotopes in precipitation unconfounded from the summer monsoon strength. Our data show, that there is a strong seasonal cycle in the isotopic composition, with low values during summer and high values during spring/winter, while the seasonal cycle in precipitation amount peaks in autumn. We find that the seasonal isotopic variability in $\delta ^{18}\hbox {O}$ and $\delta ^{2}\hbox {H}$ is controlled by a shift between two oceanic moisture sources. During summer, the Bay of Bengal is the main source location and during the remaining part of the year, the South China Sea contributes most moisture. Thus, precipitating moisture arriving in central Vietnam crosses mainland Southeast Asia only during summer. By simulating the spatial variability of $\delta ^{18}\hbox {O}$ in summer precipitation we find that rainwater isotopes across mainland Southeast Asia are mainly related to the altitude and rainout effect. Thus, both effects amplify the seasonal difference in the isotopic composition between summer and spring/autumn/winter rainfall even further.

By investigating the transition period from one source to another (once in May and again in September to November), we find that during May the relative distance of moisture uptake within the Bay of Bengal differs from year to year and thus drives interannual variability in the isotopic composition of precipitation. A similar process can be observed in September to November, where lower values in $\delta ^{18}\hbox {O}$ and $\delta ^{2}\hbox {H}$ are related to a longer travel distance of moisture within the Bay of Bengal, but also to an additional source of moisture contributed from the southern South China Sea. During years with higher mean values for September to November, this second source within the southern South China Sea is absent and moisture from the Bay of Bengal arrives from a more proximal location, close to the coast of Myanmar and Thailand. These small changes within each moisture source are governed by the timing in ITCZ migration varying per year. This means that the relative travel distance and contribution of moisture from each source is regulated by the duration in which the ITCZ remains north of central Vietnam.

We conclude that palaeoclimate archives from central Vietnam, based on stable isotopes, have the potential to capture changes in the relative moisture contribution sourced from the Indian Ocean versus the South China Sea and thus track variability in the past position of the ITCZ. Our findings suggest that a long-term northwards displacement of the ITCZ would result in more negative values in the isotopic composition of precipitation in central Vietnam and a southwards displacement in more positive values.

## Methods

### Data collection

Meteorological and water isotopic data was obtained from the International Atomic Energy Agency^44^ WISER database, which gives access to data from GNIP. The GNIP station at Dong Hoi represents central Vietnam in this study and includes five years of data from 2014 to 2018. The year of 2015 is incomplete, with March, April, May and August missing, and contains seven data points for stable isotopes. The deuterium excess was calculated following Craig^59^. Further, we use GNIP data from Yangon (Myanmar), Bangkok (Thailand), Nakorn Phanom (Thailand) and Haikou (Hainan) to compare observed data with the modeled spatial evolution of $\delta ^{18}O_p$.

### Modelling moisture uptake with PySplit

The HYSPLIT model^86^ was developed to track air parcels moving towards or from a set location. The model incorporates meteorological data and reconstructs the movement of air masses. Backward trajectories were calculated using frequency analysis with new trajectories every 6 hours for 30 days at a height of 1500 m above mean sea level, using the NCEP Global Data Assimilation System (GDAS)^87^ model one-degree archive. The flow at this level is representative of the large-scale, near surface flow in the tropics and captures the moist surface in which convergence occurs^88,89^. Moisture uptake is calculated following Sodemann et al.^90^ using PySplit^50^. Briefly, the change in humidity over 6 h periods of a HYSPLIT trajectory is calculated. When values are positive, greater than a threshold (0.2 g/kg), and the change in humidity occurs within the planetary boundary layer, it is assumed that the moisture required is taken up from surface evaporation.

### Modelling spatial evolution of $\delta ^{18}O_p$ with simple water vapor transport model

We use the Kukla et al.^91^ model to simulate the spatial evolution of oxygen isotopes, along a transect, affected by rainout effects and local topography^92^. The model calculates hydroclimatic fluxes and corresponding $\delta ^{18}O_p$ by combining three frameworks: an orographic rainout model^93^, the water balance framework^94^, and a vapour transport model^95^. This approach allows us to predict the evolution of $\delta ^{18}O_p$ by incorporating orographic precipitation and the regional water-energy budget^92^. Parameters used in this study are listed in Table 1. Initial vapour content and advective velocity are derived from the mean u and v wind fields of the ERA5 dataset^56^. Potential evapotranspiration from the CGIAR-CSI Global-Aridity and Global-PET Database^96,97^ and dryness index was calculated using GNIP data. To understand the effects of topography on oxygen isotopes, we compare a flat surface simulation (simulation 1) with a simulation incorporating local topography (simulation 2). Two mountains are used in simulation 2, represented by simple Gaussian bell shapes with peak elevation of 700 m and a width of 130 km for the Tenasserim Hills and peak elevation of 1500 m and a width of 160 km for the Truong Son Mountains. Both simulations use the same climatic parameters (Table 1), which are representing typical conditions during the period of June to August. Moisture arriving in central Vietnam only crosses landmasses during this time of the year, thus allowing us to investigate continental rainout and altitude effects.

**Table 1:** Model input used in the simulations 1 and 2 for the period of June to August.

Nodes	500
Length of domain	$3.0^6$ (m)
Elevation of Truong Son and Tenasserim Hills	1500 and 700 (m)
Mean annual temperature (2 m above sea level)^44^	303 (K)
Potential evapotranspiration^97^	556 (mm)
Péclet number^98^	30
Initial vapour content^56^	60 (kg m$^{-2}$)
Relative humidity^99^	0.8
Advective velocity^56^	3 (m s$^{-1}$)
Transpired fraction of evapotranspiration^100^	0.64
Starting dryness index^97^	2.37
Recycling efficiency parameter (global mean)^92^	2.6

Initial vapour content and advective velocity are derived from the ERA5 dataset^56^. Potential evapotranspiration from the CGIAR-CSI Global-Aridity and Global-PET Database^96,97^ and dryness index was calculated using GNIP data.

## Data Availability

All data used for this research are available in the methods and at: GNIP isotope data is accessible at http://www.iaea.org/water. GPCC Precipitation, Interpolated OLR and NCEP Global Data Assimilation System data were provided by the NOAA/OAR/ESRL PSD, Boulder, Colorado, USA, from their Web site at https://psl.noaa.gov/. The ERA5 database can be accessed at https://cds.climate.copernicus.eu/cdsapp#!/home. The annual tropical cyclone reports can be access at: https://www.usno.navy.mil/JTWC/annual-tropical-cyclone-reports.
